# Electroacupuncture Improves Cerebral Vasospasm and Functional Outcome of Patients With Aneurysmal Subarachnoid Hemorrhage

**DOI:** 10.3389/fnins.2018.00724

**Published:** 2018-10-09

**Authors:** Jie Sun, Yuchun Liu, Junjun Zhang, Xiaosheng Chen, Zhiqing Lin, Sheng Nie, Manhua Shi, Xiang Gao, Yi Huang

**Affiliations:** ^1^Department of Neurosurgery, Ningbo First Hospital, Ningbo Hospital, Zhejiang University School of Medicine, Ningbo, China; ^2^Department of Acupuncture, Ningbo First Hospital, Ningbo Hospital, Zhejiang University School of Medicine, Ningbo, China

**Keywords:** aneurysmal subarachnoid hemorrhage, electroacupuncture, cerebral vasospasm, Baihui (GV20), computed tomographic perfusion, transcranial doppler

## Abstract

Cerebral vasospasm is the major cause of a poor outcome after aneurysmal subarachnoid hemorrhage (aSAH), and effective treatments for vasospasm are limited. The purpose of this study was to research the impact of electroacupuncture (EA) on cerebral vasospasm and the outcomes of patients with aSAH. A total of 60 age- and sex-matched aSAH patients were collected from Ningbo First Hospital between December 2015 and June 2017. All patients were given a basic treatment of nimodipine and randomized into two groups. The study group was treated with EA therapy on the Baihui (GV20) acupoint, and the control group was given mock transcutaneous electrical nerve stimulation. Cerebral vasospasm was measured by computed tomographic perfusion (CTP) and transcranial doppler (TCD). The mean flow velocity (MFV) in the middle cerebral artery (MCA), cerebral blood flow (CBF), cerebral blood volume (CBV), and mean transit time (MTT) of the patients were analyzed. The CBV and MTT exhibited significant differences between the study and control groups on the 1st (*p* = 0.026 and *p* = 0.001), 7th (*p* = 0.020 and *p* < 0.001), and 14th (*p* = 0.001 and *p* < 0.001) day after surgery, whereas CBF exhibited statistical significance only on the 14th day after surgery (*p* = 0.002). The MFV in MCA were significantly reduced after EA treatment in all patients (all *p* < 0.001). Additionally, the MFV in the MCA in patients treated with EA were considerably reduced compared with those of the control group (3rd day *p* = 0.046; 5th day, *p* = 0.010; 7th day, *p* < 0.001). Moreover, better outcomes were noted in the EA-treated group for the 1st month (*p* < 0.001) and 3rd month (*p* = 0.001) after surgery than in the control group. In conclusion, EA represents a potential method to treat cerebral vasospasm after aSAH and can improve the outcomes of patients with aSAH.

## Introduction

Aneurysmal subarachnoid hemorrhage (aSAH) is a devastating event accounted for 5% of all stroke cases (Sehba et al., 2012; Garg and Bar, 2017). The incidence of aSAH is approximately 9 per 100,000, and the mortality is approximately 60% within 6 months (Steiner et al., 2013). The surgical treatment of aSAH includes endovascular coiling and surgical clipping. However, there are a lot of complications frequently occur after successful surgery (Connolly et al., 2012; Steiner et al., 2013), such as cerebral vasospasm, rebleeding, or hydrocephalus. Cerebral vasospasm typically appears on the third day after aSAH, is maximal at 6–8 days, and subsequently lasts 2–3 weeks (Wilkins, 1990). There are approximately 23% of deaths or disabilities in patients with aSAH due to cerebral vasospasm (Keyrouz and Diringer, 2007). Delayed cerebral vasospasm is considered as the major cause of poor outcomes in aSAH patients in subsequent decades (Dusick and Gonzalez, 2013).

According to the past research, cerebral vasospasm after aSAH was regarded as an outcome of many factors, such as inflammatory changes, abnormal endothelial hypertrophy, prolonged contraction of the smooth muscle cells, and gene expression in the brain arteries (Rothoerl and Ringel, 2007). In addition, it seems possible that disruptions in either or both nitric oxide and endothelin-1 metabolism may play key roles in the pathogenesis of cerebral vasospasm (Findlay et al., 2016). Consequently, various methods are used to prevent cerebral vasospasm on the basis of its pathogenesis, such as the endothelin antagonist clazosentan, the cholesterol-lowering agent simvastatin, and the vasodilator magnesium sulfate, calcium channel blocker nicardipine (van Gijn and Rinkel, 2001; Wilson et al., 2005; van Gijn et al., 2007). However, some researchers find that the prevention of cerebral vasospasm does not improve the outcomes of aSAH patients (Macdonald et al., 2008; Vergouwen, 2009; Macdonald et al., 2011).

Acupuncture has been used to treat aSAH effectively and safely for centuries (Hu et al., 1993). In recent years, numerous studies have demonstrated that acupuncture can increase the content of nitric oxide in vascular endothelial cell, activate the activity, and improve the function of injured vascular endothelial cells (Chen and Ma, 2003; Kim et al., 2006). In addition, acupuncture or EA at the Baihui (GV20) and left Zusanli (ST36) acupoints in rat model significantly reduces the expression of the proinflammatory enzyme MMP2 and the water channel proteins AQP4, to relieve inflammation related brain edema (Xu et al., 2014). Therefore, we hypothesized that EA might be used to improve cerebral vasospasm and the outcomes of patients with aSAH. In this study, we recruited 60 age- and sex-matched volunteers and performed a case–control study to investigate and validate the impact of EA treatment on patients with aSAH from a Chinese Han population.

## Materials and Methods

### Participants

This double-blinded, randomized controlled study was approved by the Ethics Committee of Ningbo First Hospital. A total of 60 age- and sex-matched aSAH patients from Department of Neurosurgery, who were randomly allocated by computer algorithm to the study group (*n* = 30) or the control group (*n* = 30). Inclusion criteria were as follows: (1) the individuals were aged older than 18 years and had the ability to begin acupuncture treatment within 24 h after aSAH; (2) aSAH were independently diagnosed by at least two neurosurgeons using computed tomography and cerebral angiography; (3) the patients with aneurysm who had been treated by the operative treatment such as endovascular coiling or surgical clipping; (4) written informed consent was obtained before the study. Exclusion criteria were as follows: (1) the patients with traumatic or infectious aSAH; (2) the patients without ability to undergo TCD; (3) the patients with severe heart, hepatic and renal dysfunctions; (4) the patients with cardiac pacemaker; or (5) the patients with a previous EA experience.

### Intervention

EA treatment started within 24 h of aSAH, after intracranial aneurysm surgery including endovascular coiling or surgical clipping. EA was applied four times in 2 weeks, and it was performed by the same acupuncturist with over 10 years acupuncture treatment experience. At the same time, standard medicine treatments, such as hemodilution (triple-H) therapy, nimodipine, hypertension control, and hypervolemic, were appropriated for all patients.

### Study and Control Group

**FIGURE 1:**
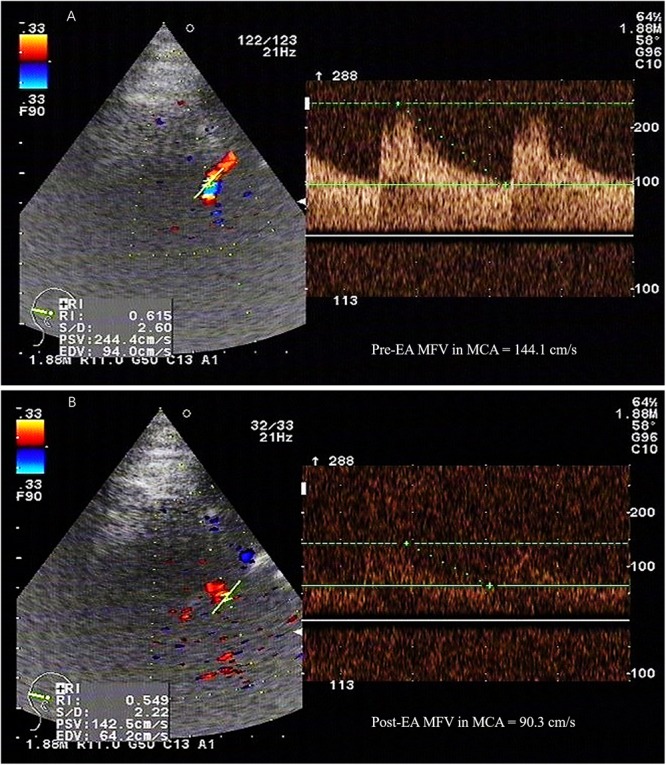
The MFV in the MCA was measured by TCD. PSV, peak systolic velocity; EDV, end-diastolic velocity; MFV, mean flow velocity; MCA, middle cerebral artery; TCD, transcranial doppler; EA, electroacupuncture. A 52-year-old female patient with subarachnoid hemorrhage caused by rupture of right anterior communicating artery aneurysm, developed vasospasm on the 5th day after surgery. The maximum value of flow velocity in systole was calculated at the apex of the waveform, which defined as PSV. EDV was measured at end diastole, and indicated the lowest point of waveform. MFV was calculated as EDV plus a third of the difference between PSV and EDV (MFV [cm/s] = [PSV + 2EDV]/3). TCD detection showed that the MFV in MCA in the range of 120∼140 cm/s was indicative of mild cerebral vasospasm, 140∼200cm/s was accord with moderate cerebral vasospasm, and above 200 cm/s was consistent with severe cerebral vasospasm. **(A)** TCD showed a high MFV in MCA (144.1cm/s) before EA. **(B)** The MFV in MCA (90.3cm/s) was decreased after EA.

The patients in the study group were administered EA therapy using HANS100A analgesic apparatus (Nanjing Jisheng Medical Technology Co., Nanjing, China) with a dilatational wave of 2 Hz in the GV20 position. The electric current was gradually increased (0 up to 5 mA), and the speed depended on the patient’s pain threshold. Each treatment session based on the electric current intensity of 5 mA and lasted 30 min. EA treatment was performed on the 1st, 3rd, 5th, and 7th days after the operation. The MFV in the MCA was measured by TCD (Prosound α10, Aloka Co, Ltd, Tokyo, Japan) before and after EA therapy (**Figure 1**). The MFV was calculated according to the primary study (Lysakowski et al., 2001). The control group received standard medicine treatments as same as the study group, but without EA treatment. In addition, cerebral vasospasm was measured by CTP on the 1st, 7th, and 14th day after surgery, and the dates of CBF, CBV, and MTT were collected.

### Statistics

The sample size was calculated based on the primary study. The incidence rate of delayed ischemic neurological deficit in the acupuncture group was 10%, and the control group was 38.9% (Cho et al., 2015). To detect a significance level of 0.05 between the groups with 80% power at the assumption of a drop-out rate of 20% and a 1-tailed α = 0.05, 54 patients (27 participants in each group) were required for this study (Shuster, 2014). Data were represented as mean ± standard deviation (SD) or number (%) and analyzed using SPSS version 23.0 (IBM Corp. Armonk, NY, United States) software. Categorical variables were analyzed with the chi-square test or Fisher’s exact test, and the means of continuous variables were analyzed using the Student’s *t*-test. Statistical significance was defined as a *p*-value of <0.05.

## Results

### Clinical Characteristics of the Subjects

The clinical characteristics of the patients are presented in **Table 1**. The control group consisted of 30 aSAH patients (13 males and 17 females) with a mean age of 51.9 ± 9.51 years. The study group underwent EA therapy and consisted of 30 aSAH patients (13 males and 17 females) with a mean age of 52.67 ± 11.27 years. No significant differences in the aneurysm location (*p* = 0.612), aneurysms therapies (*p* = 0.121), Hunt-Hess grade (*p* = 0.793), and Fisher grade (*p* = 0.279) were noted between the two groups.

**Table 1:** Patient characteristics of the two groups.

Characteristics	Study group (*n* = 30)	Control group (*n* = 30)	*p*-value
Age(years)	52.67 ± 11.27	51.9 ± 9.51	0.777
Male/female	13/17	13/17	1.000
Aneurysm location			0.612
Anterior communicating Artery	13	13	
Posterior communicating Artery	7	10	
Middle cerebral artery	2	3	
Posterior inferior cerebellar artery	3	0	
Basilar artery	0	1	
Vertebral artery	2	1	
Supraclinoid internal carotid artery	2	1	
Multiple intracranial aneurysms	1	1	
Aneurysms therapies			0.121
Coiling	4	26	
Clipping	0	30	
Hunt–Hess grade			0.793
I–II	17	18	
III–V	13	12	
Fisher grade			0.279
I	7	12	
II-III	19	13	
IV	4	5	


*Age was analyzed using student’s *t*-test and other data were analyzed using chi-square test.*

### Cerebral Vasospasm Was Measured by CTP

**FIGURE 2:**
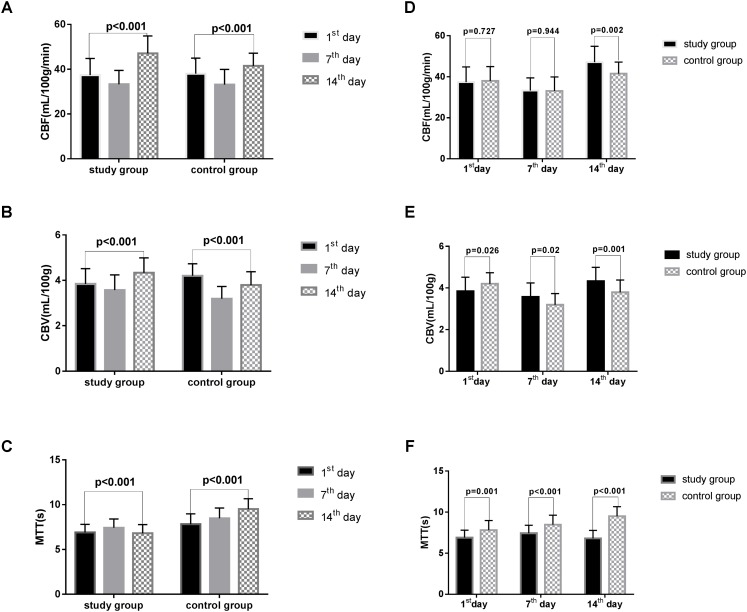
The CBF, CBV, MTT in two groups. CBF, cerebral blood flow; CBV, cerebral blood volume; MTT, mean transit time. All the data were analyzed using student’s *t*-test. **(A)** CBF variations in the two groups based on time after surgery. **(B)** The variation in CBV in the two groups based on time after surgery. **(C)** The variation in the MTT of the two groups based on time after surgery. **(D)** The difference in CBF in two groups on the 1^st^, 7^th^, and 14^th^ day after surgery. **(E)** The difference in CBV in the two groups on the 1^st^, 7^th^, and 14^th^ day after surgery. **(F)** The difference in MTT in the two groups on the 1^st^, 7^th^, and 14^th^ day after surgery.

Significant differences in CBF, CBV, and MTT variations were noted in both groups on the 1st, 7th, and 14th days after surgery (all *p* < 0.001, **Table 2**, **Figures 2A–C**). The CBF was considerably increased in the study group on the 14th day after surgery (study group vs. control group: 47.10 ± 7.79 vs. 41.52 ± 5.65, *p* = 0.002, **Table 3**, **Figure 2D**). The CBV was markedly increased in the study group compared with the control group on the 1st (study group vs. control group: 3.84 ± 0.68 vs. 4.20 ± 0.53, *p* = 0.026), 7th (study group vs. control group: 3.57 ± 0.67 vs. 3.19 ± 0.54, *p* = 0.020), and 14th days (study group vs. control group: 4.33 ± 0.66 vs. 3.79 ± 0.59, *p* = 0.001, **Table 3**, **Figure 2E**) after surgery. Besides, the MTT was considerably reduced in the study group compared with the control group on the 1st (study group vs. control group: 6.89 ± 0.92 vs. 7.82 ± 1.16, *p* = 0.001), 7th (study group vs. control group: 7.43 ± 0.98 vs. 8.48 ± 1.15, *p* < 0.001), and 14th (study group vs. control group: 6.81 ± 0.97 vs. 9.50 ± 1.17, *p* < 0.001, **Table 3**, **Figure 2F**) days after surgery.

**Table 2:** The changes in CBF, CBV, and MTT in the two groups over time.

	Variable	1^st^ day	7th day	14th day	*p*-value
	CBF (mL/100 g/min)	37.40 ± 7.44	33.26 ± 6.20	47.10 ± 7.79	<0.001
Study group	CBV (mL/100 g)	3.84 ± 0.68	3.57 ± 0.67	4.33 ± 0.66	<0.001
	MTT (s)	6.89 ± 0.92	7.43 ± 0.98	6.81 ± 0.97	<0.001
	CBF (mL/100 g/min)	38.05 ± 6.94	33.15 ± 6.76	41.52 ± 5.65	<0.001
Control group	CBV (mL/100 g)	4.20 ± 0.53	3.19 ± 0.54	3.79 ± 0.59	<0.001
	MTT (s)	7.82 ± 1.16	8.48 ± 1.15	9.50 ± 1.17	<0.001


*CBF, cerebral blood flow; CBV, cerebral blood volume; MTT, mean transit time.*

*All the data were analyzed using student’s *t*-test.*

**Table 3:** Comparisons of CBF, CBV, and MTT in the two groups over time.

Variable	Study group	Control group	*p*-value
CBF1^st^ (mL/100 g/min)	37.40 ± 7.44	38.05 ± 6.94	0.727
CBF7^th^ (mL/100 g/min)	33.26 ± 6.20	33.15 ± 6.76	0.944
CBF14^th^ (mL/100 g/min)	47.10 ± 7.79	41.52 ± 5.65	0.002
CBV1^st^ (mL/100 g)	3.84 ± 0.68	4.20 ± 0.53	0.026
CBV7^th^ (mL/100 g)	3.57 ± 0.67	3.19 ± 0.54	0.020
CBV14^th^ (mL/100 g)	4.33 ± 0.66	3.79 ± 0.59	0.001
MTT1^st^ (s)	6.89 ± 0.92	7.82 ± 1.16	0.001
MTT7^th^ (s)	7.43 ± 0.98	8.48 ± 1.15	<0.001
MTT14^th^ (s)	6.81 ± 0.97	9.50 ± 1.17	<0.001


*CBF1st/7th/14th, cerebral blood flow on the 1^*st*^, 7^*th*^, and 14^*th*^ day after surgery, respectively; CBV1st/7th/14^*th*^, cerebral blood volume on the 1^*st*^, 7^*th*^, and 14^*th*^ day after surgery, respectively; MTT1st/7^*th*^/14^*th*^, mean transit time on the 1^*st*^, 7^*th*^, and 14^*th*^ day after surgery, respectively. All the data were analyzed using student’s *t*-test.*

### Cerebral Vasospasm Was Relieved After EA Therapy and Measured by TCD

As presented in **Table 4**, statistically significant variations in the MFV levels of MCA were noted between the two groups based on the length of time after surgery. Significant differences in the MFV of MCA were noted between the two groups on the 3rd (study group vs. control group: 115.32 ± 19.22 vs. 124.60 ± 15.80, *p* = 0.046), 5th (study group vs. control group: 117.34 ± 18.49 vs. 128.68 ± 14.37, *p* = 0.010), and 7th (study group vs. control group: 108.19 ± 16.16 vs. 124.94 ± 14.85, *p* < 0.001, **Table 4**, **Figure 3A**) day after surgery. In the study group, all patients accepted EA therapy at the same time point after surgery (1st, 3rd, 5th, and 7th day), and the MFV in MCA was significantly reduced compared with that measured post-EA on the 1st (Pre-EA vs. Post-EA: 118.49 ± 20.38 vs. 109.12 ± 18.40, *p* < 0.001), 3rd (Pre-EA vs. Post-EA: 125.29 ± 22.51 vs. 115.32 ± 19.22, *p* < 0.001), 5th (Pre-EA vs. Post-EA: 128.37 ± 22.15 vs. 117.34 ± 18.49, *p* < 0.001), and 7th (Pre-EA vs. Post-EA: 118.94 ± 18.61 vs. 108.19 ± 16.16, *p* < 0.001, **Table 5**, **Figure 3B**) day.

**Table 4:** The MFV level in MCA in the two groups.

Time day	Study group	Control group	*p*-value
1^st^ (cm/s)	109.12 ± 18.40	117.42 ± 17.67	0.080
3^rd^ (cm/s)	115.32 ± 19.22	124.60 ± 15.80	0.046
5^th^ (cm/s)	117.34 ± 18.49	128.68 ± 14.37	0.010
7^th^ (cm/s)	108.19 ± 16.16	124.94 ± 14.85	<0.001
*p-*value	<0.001	<0.001	


*MFV, mean flow velocity; MCA, middle cerebral artery. All the data were analyzed using student’s *t*-test.*

**FIGURE 3:**
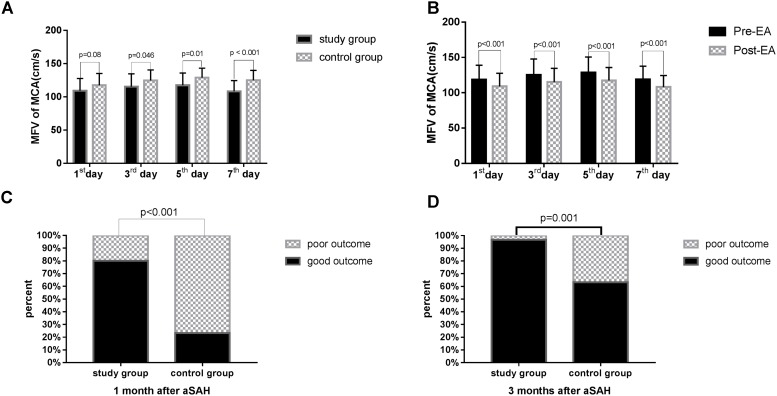
The MFV in MCA and the outcome in the two groups. MFV, mean flow velocity; MCA, middle cerebral artery. The GOS score was used 1 or 3 months after surgery to assess the clinical outcome of patient survival. **(A)** Comparison of MFV in MCA in the study group versus control group on the 1^st^, 3^rd^, 5^th^, and 7^th^ day after surgery. The data were analyzed using student’s *t*-test. **(B)** Comparison of MFV in MCA in the study group pre-EA versus post-EA on the 1^st^, 3^rd^, 5^th^, and 7^th^ day after surgery. The data were analyzed using student’s *t*-test. **(C)** Comparison of outcome in the study group versus control group 1 month after surgery The data were analyzed using chi-square test. **(D)** Comparison of outcome in the study group versus control group 3 months after surgery. The data were analyzed using chi-square test.

**Table 5:** The MFV level in MCA in the study group.

	MFV1^st^	MFV3^rd^	MFV5^th^	MFV7^th^
Pre-EA (cm/s)	118.49 ± 20.38	125.29 ± 22.51	128.37 ± 22.15	118.94 ± 18.61
Post- EA (cm/s)	109.12 ± 18.40	115.32 ± 19.22	117.34 ± 18.49	108.19 ± 16.16
*p*-value	<0.001	<0.001	<0.001	<0.001


*EA, electroacupuncture; MFV, mean flow velocity; MCA, middle cerebral artery; MFV1^*st*^/3^*rd*^/5^*th*^/7^*th*^, mean flow velocity in the study group on the 1^*st*^, 3^*rd*^, 5^*th*^, and 7^*th*^ day after the operation.*

*All the data were analyzed using student’s t-test.*

### Patients With aSAH Exhibited a Better Outcome After EA Treatment

The GOS score was used 1 or 3 months after surgery to assess the clinical outcome of patient survival. Significant differences were noted in the outcomes of the two groups at 1 (*p* < 0.001, **Table 6**, **Figure 3C**) and 3 months (*p* = 0.001, **Table 6**, **Figure 3D**) after surgery, and more patients exhibited a good outcome in the study group.

**Table 6:** The outcome of aSAH patients in the two groups.

	1 Month after aSAH			3 Months after aSAH			
	Good outcome (GOS > 3)		Poor outcome (GOS≦a3)		Good outcome (GOS > 3)		Poor outcome (GOS≦3)
Study group	24		6		29		1
Control group	7		23		19		11
*p*-value		<0.001				0.001	


*aSAH, Aneurysmal subarachnoid hemorrhage; GOS, Glasgow Outcome Scale. All the data were analyzed using chi-square test.*

## Discussion

Cerebral ischemia after aSAH is a complex complication involving delayed cerebral vasospasm that may cause clinical deterioration, cerebral infarctionand death. Cerebral vasospasm after aSAH may related with multifactorial etiology, and this hypothesis was confirmed by the massive available treatment modalities. Currently, strong evidence supports that nimodipine and the triple-H therapy are benefits for cerebral vasospasm patients (Adamczyk et al., 2013). Many treatments have improved the prognosis, but the neurological outcomes remain poor in the patients with delayed cerebral vasospasm. Therefore, it is urgent to identify safer and more efficacious methods to manage cerebral vasospasm and improve the outcomes of aSAH patients. EA is a advanced therapy on the basis of combination of traditional acupuncture and modern electrotherapy and is recommended as a treatment for stroke (Li et al., 2014). Here, our results demonstrate that EA at GV20 significantly reduces cerebral vasospasm after aSAH and improves the outcomes of aSAH patients.

Acupuncture at the Baihui (GV20) acupoint had been used to treat stroke for hundreds of years (Wang et al., 2014). Currently, scientists revealed that acupuncture Baihui (GV20) could protect the neuro in ischemic stroke animal models. Dong et al. (2009) study suggested that EA preconditioning of the Baihui (GV20) acupoint could ease the brain edema and attenuate blood–brain barrier disruption via effecting the related protein activity. Kim et al. (2013) found that EA at the Baihui (GV20) and Dazhui (GV14) acupoints could increase CBF and improve the functional recovery in acute moderate focal cerebral ischemia. Wang et al. (2012) reported that EA pretreatment at the Baihui (GV20) acupoint could protect the brain against transient ischemic injury through activating the anti-inflammatory proteins. Given that cerebral vasospasm begins 24–72 h after aSAH, early intervention of EA is urgent and necessary. In our study, in all patients who accepted EA therapy after coiling or clipping, the occurrence of rebleeding during EA treatment was avoided. In addition, to ensure the safety of all patients, treatment with nimodipine and other supporting agents was also administered. There are various advantages of EA compared with manual acupuncture. EA can be performed standardly, measured objectively, and controlled fully. Besides, EA has also been recommended for clinical trials and mechanistic research on acupuncture (Tsuchiya et al., 2007).

In our study, cerebral vasospasm was monitored by CTP and TCD. CTP has been used in patients with aSAH (Sviri et al., 2006; van der Schaaf et al., 2006) and has rapidly gained popularity given its simplicity, speed, and veracity (Washington et al., 2011). CTP can detect the reduction of CBF, prolongation of MTT, and the status of CBV (Dankbaar et al., 2010). Moreover, threshold values of 35 mL/100 g/min for CBF and 5.5 s for MTT are suggested for the diagnosis of cerebral vasospasm (Sanelli et al., 2011), and CBV exhibited the lowest diagnostic accuracy among all variables (Othman et al., 2016). The results of this study demonstrated that the CBV and MTT were significantly different between the study and control groups at 1, 7, and 14 days after surgery, whereas CBF exhibited statistical significance only 14 days after surgery. In addition, TCD works on that blood-flow velocity within an artery increases as the artery narrows, result in doppler frequency shift (Purkayastha and Sorond, 2012). The maximum value of flow velocity in systole was calculated at the apex of the waveform, which defined as PSV (Fotakopoulos et al., 2018). EDV was measured at end diastole, and indicated the lowest point of waveform (Fotakopoulos et al., 2018). MFV was calculated as EDV plus a third of the difference between PSV and EDV (MFV [cm/s] = [PSV + 2EDV]/3) (Lysakowski et al., 2001). TCD detection showed that the MFV in MCA in the range of 120 ∼ 140 cm/s was indicative of mild cerebral vasospasm, 140 ∼ 200 cm/s was accord with moderate cerebral vasospasm, and above 200 cm/s was consistent with severe cerebral vasospasm (Sebastian et al., 2013). A skilled researcher operated TCD monitoring at a specific time every other day. In our study, the MFV levels of MCA were significantly reduced after EA treatment in the same patients. Additionally, the MFV levels of MCA in patients with EA treatment were markedly reduced than the control group. These findings suggest that EA is a potential method to relieve cerebral vasospasm after aSAH. Recent study suggested that EA Baihui (GV20) and Dazhui (GV14) could significantly increase cerebral cortex acetylcholine release, and repair nerve damage (Kim et al., 2013). Several studies have demonstrated that acupuncture enhances the nitric oxide production and increases local circulation (Tsuchiya et al., 2007). Dong et al. (2009) found that EA at GV20 could reduce MMP2 and the water channel proteins, AQP4 and AQP9, which repair brain injury and improve functional outcomes depending on the mitigation of inflammation-related brain edema in model rats. We believe that these effects may explain the main mechanism through which EA treatment improves patient outcomes, and the outcome of the study group was significantly improved than the control group. Therefore, we think that the effect of EA treatment appears after it is continuously applied to aSAH patients over a 2-week period.

### Study Limitations

Apparently, our research has several limitations. First, only one center was included in this study, multicenter with a more homogeneous samples were needed to certificate the specific treatment effect in the future. Second, only a limited number of measurement timepoints were included in the study, so more frequent timepoints in the assessment of cerebral vasospasm should be included in future studies. Third, protein signaling pathways were not investigated in the current study, but should be a focus of future research. Fourth, the craniotomy procedure might affect cerebral vasospasm and the curative effect of EA therapy. Thus, a comparison between the clipping and coiling group is required in the future.

## Conclusion

In summary, our study confirmed that cerebral vasospasm was presented in patients with aSAH. EA therapy at the Baihui (GV20) position could increase the MFV of cerebral circulation and improve the outcome of aSAH patients. It is a safe and convenient treatment to improve cerebral vasospasm and the outcomes of aSAH patients.

## Author Contributions

XG, JS, and YH designed the study and edited the manuscript. ZL, YL, JZ, SN, and MS were responsible for data acquisition and experiments. YL and XC conducted data analysis and drafted the manuscript.

## Conflict of Interest Statement

The authors declare that the research was conducted in the absence of any commercial or financial relationships that could be construed as a potential conflict of interest.

## Abbreviations

AQP: aquaporin
aSAH: aneurysmal subarachnoid hemorrhage
CBF: cerebral blood flow
CBV: cerebral blood volume
CBV: cerebral blood volume
CTP: computed tomographic perfusion
EA: electroacupuncture
EDV: end-diastolic velocity
GOS: glasgow outcome scale
MCA: middle cerebral artery
MFV: mean flow velocity
MMP: matrix metalloproteinase
MTT: mean transit time
PSV: peak systolic velocity
TCD: transcranial doppler
